# Editorial Board

**DOI:** 10.1080/23802359.2016.1272841

**Published:** 2016-12-23

**Editor-in-Chief**

Rob DeSalle - American Museum of Natural History, 79th Street at Central Park W, New York, NY 10024, USA. http://desalle.amnh.org

**Associate Editor**

Mikhail Alexeyev - University of South Alabama, Dept. Physiology and Cell Biology, 5851 USA Dr. North, MSB3074, Mobile, AL 36688, USA

**Managing & Handling Editor**

Sergios-Orestis Kolokotronis - SUNY Downstate Medical Center, School of Public Health, 450 Clarkson Avenue, Mail Stop Code 43, Brooklyn, NY 11203-2098, USA

